# Supplementary material for: Fish heating tolerance scales similarly across individual physiology and populations
Source: Commun Biol. 2021 Mar 1;4:264. doi: 10.1038/s42003-021-01773-3 (PMC7921436; doi:10.1038/s42003-021-01773-3)
Supplement: Supplementary file 2 — Supplementary Information [file 42003_2021_1773_MOESM2_ESM.pdf]

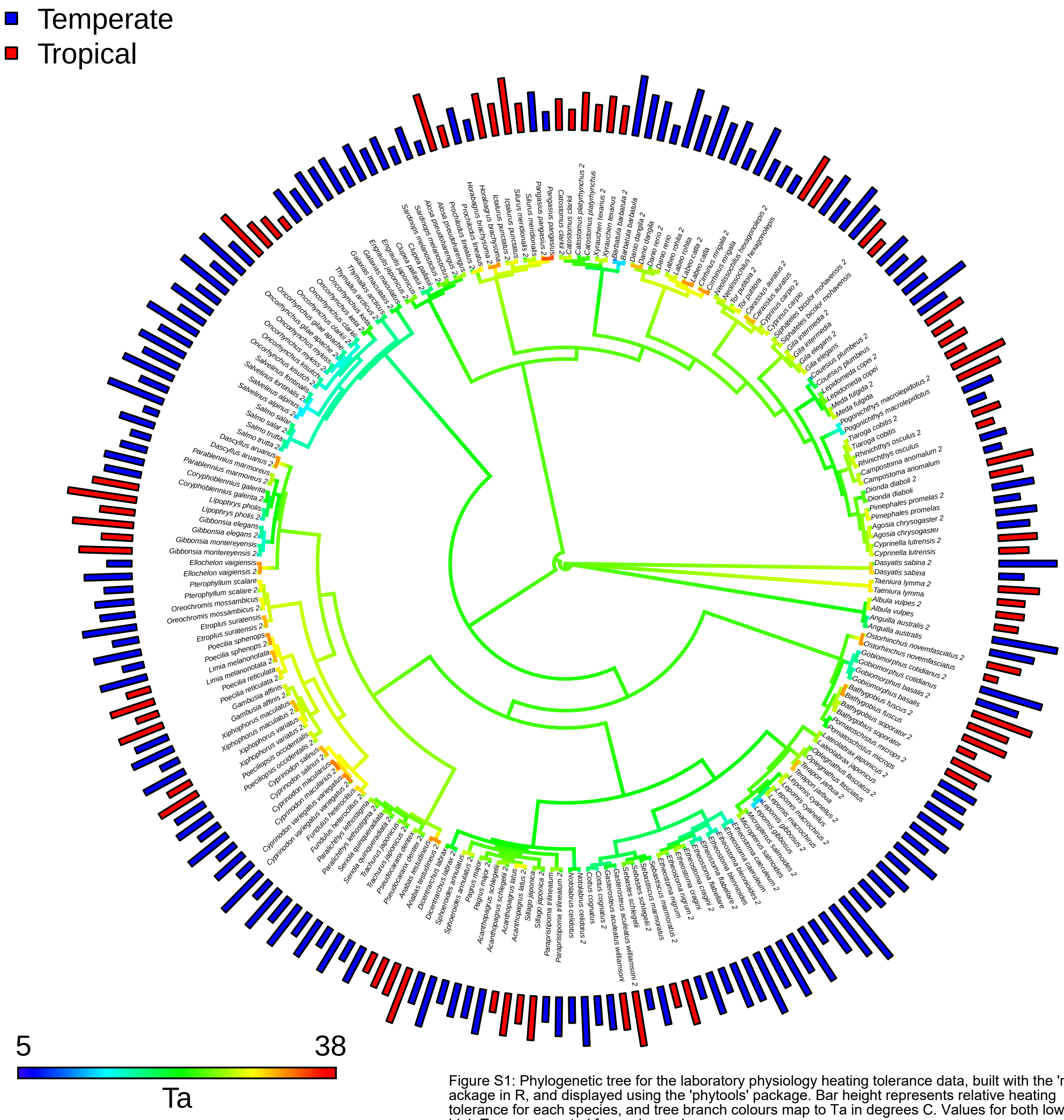

Figure S1: Phylogenetic tree for the laboratory physiology heating tolerance data, built with the 'rotl' package in R, and displayed using the 'phytools' package. Bar height represents relative heating tolerance for each species, and tree branch colours map to Ta in degrees C. Values for both low and high Ta are presented for each species.

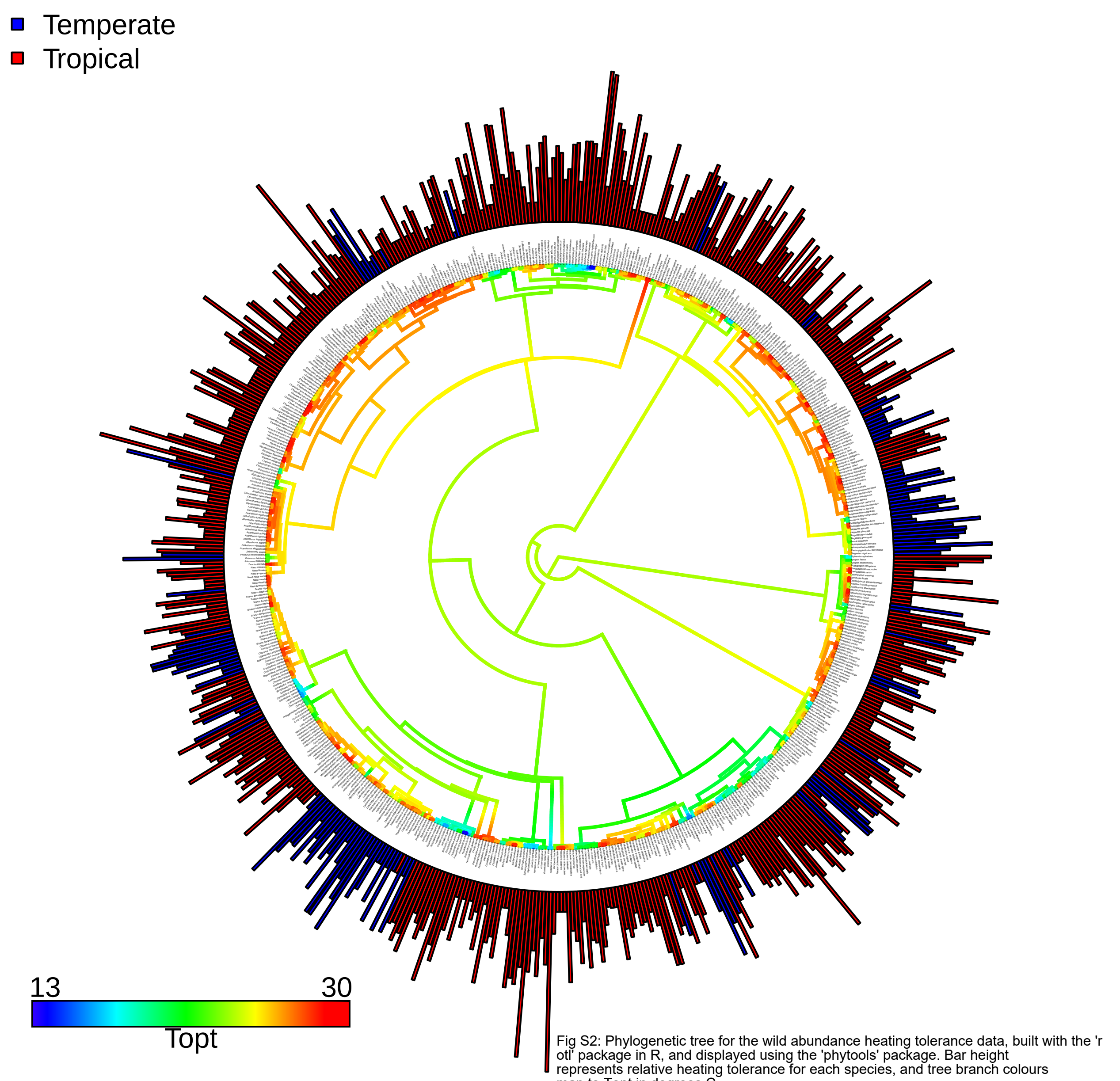

Fig S2: Phylogenetic tree for the wild abundance heating tolerance data, built with the 'rotl' package in R, and displayed using the 'phytools' package. Bar height represents relative heating tolerance for each species, and tree branch colours map to Topt in degrees C.

Supplementary Table 1. Results of linear model for heating tolerance of individual physiology. Effects are for acclimation temperature T_a, thermal guild (tropical or temperate) and relative T_a (high or low). Polar species have insufficient data for relative T_a so are excluded from the model. The model has adjusted R^2 of 0.73 and F of 112.0 on 208 degrees of freedom.

	Estimate	Std. Error	t value	Pr(>|t|)
(Intercept)	18.98	1.28	14.87	<0.001
Ta	-0.36	0.05	-7.42	<0.001
guildtrop	5.06	1.92	2.63	0.009
relative_Talow Ta	4.44	1.43	3.10	0.002
Ta:relative_Talow Ta	-0.11	0.06	-1.82	0.070
Ta:guildtrop	-0.12	0.07	-1.76	0.080

Supplementary Table 2. Results of PGLS for heating tolerance of individual physiology. Effects are for acclimation temperature T_a, thermal guild (tropical or temperate) and relative T_a (high or low). Polar species have insufficient data for relative T_a so are excluded from the model. The model has adjusted R^2 of 0.92 and F of 494.8 on 208 degrees of freedom, and lambda of 0.994 [95% CI: 0.98-1.00].

	Estimate	Std. Error	t value	Pr(>|t|)
(Intercept)	26.31	3.90	6.75	<0.001
Ta	-0.58	0.04	-14.79	<0.001
guildtrop	1.61	1.04	1.55	0.122
relative_Talow Ta	1.36	0.73	1.85	0.065
Ta:relative_Talow Ta	-0.03	0.03	-1.24	0.216
Ta:guildtrop	-0.05	0.04	-1.30	0.196

Supplementary Table 3. Results of linear model for heating tolerance of wild populations. Effects are for T_{opt} and thermal guild (tropical or temperate). The model had adjusted R^2 of 0.86 and F of 1226 on 607 degrees of freedom.

	Estimate	Std. Error	t value	Pr(>|t|)
(Intercept)	15.53	0.53	29.32	<0.001 ***
topt	-0.57	0.03	-20.64	<0.001 ***
guildtrop	10.65	0.68	15.56	<0.001 ***
topt:guildtrop	-0.27	0.03	-8.67	<0.001 ***

Supplementary Table 4. Results of PGLS for heating tolerance of wild populations. Effects are for T_{opt} and thermal guild (tropical or temperate). The model had adjusted R^2 of 0.86, an F of 1226 on 607 degrees of freedom, and a lambda of 0.00 [upper value of 95% CI: 0.03].

	Estimate	Std. Error	t value	Pr(>|t|)
(Intercept)	15.53	0.53	29.32	<0.001 ***
topt	-0.57	0.03	-20.64	<0.001 ***
guildtrop	10.65	0.68	15.56	<0.001 ***
topt:guildtrop	-0.27	0.03	-8.67	<0.001 ***
