# Supplementary material for: Fish heating tolerance scales similarly across individual physiology and populations
Source: Commun Biol. 2021 Mar 1;4:264. doi: 10.1038/s42003-021-01773-3 (PMC7921436; doi:10.1038/s42003-021-01773-3)
Supplement: Supplementary file 3 — Description of Additional Supplementary Files [file 42003_2021_1773_MOESM3_ESM.pdf]

Description of Additional Supplementary Files

File name: Supplementary Data 1.

Description: Heating tolerance data for fishes at the individual and population levels.
